# Providing online STEM workshops in times of isolation

**DOI:** 10.1007/s43545-021-00110-z

**Published:** 2021-04-26

**Authors:** Robert Weinhandl, Susanne Thrainer, Zsolt Lavicza, Tony Houghton, Markus Hohenwarter

**Affiliations:** Linz School of Education, Johannes Kepler University, Altenberger Straße 54, 4040 Linz, Austria

**Keywords:** STEM workshops, Online learning, Non-formal learning environments, TPACK for STEM teachers

## Abstract

The global spread of COVID-19 has resulted in learning and teaching being confronted with immense challenges and changes since spring 2020. Measures to contain the COVID-19 pandemic had and continue to have a particularly strong impact on non-formal and informal learning, which are important features of out-of-class on-line STEM workshops combining science, technology, engineering and mathematics. We developed and carried out online STEM workshops for approximately 250 students during times of isolation in the spring and summer of 2020. To identify potential success factors and stumbling blocks for designing and implementing online STEM workshops, we conducted a qualitative interview-based study with a selection of eight experts in the summer and autumn 2020, i.e. after the STEM workshops. The experts were Austrian and German teachers who planned and implemented STEM workshops for students age 10 to 18. Our collected data was examined using techniques of grounded theory approaches. Using techniques of qualitative interview studies and grounded theory approaches, we found that removing learning barriers and creating new types of learning spaces, online socio-constructivist learning, and teachers' TPACK for STEM Workshops are important factors when considering and designing STEM workshops for online learning environments. These same factors will also be central when planning and implementing online STEM workshops in post-COVID-19 times. To facilitate student participation and to increase social interaction are critical elements for practitioners working on online STEM workshops—both during and after COVID-19. Highly trained teachers with in-depth technical, pedagogical, and content skills are essential to facilitate participation and interactions among students, teachers, and learning contents.

## Introduction

Learning and teaching in general, and STEM in particular, suddenly turned upside-down in Spring 2020 affecting teachers and students of all ages across the globe. Homeschooling or online learning became one of the most widespread approaches to learning during this time and, since COVID-19 vaccine development and then the global distribution of this vaccine could take months or years (Doucet et al. 2020; Prompetchara et al. 2020), there is a high chance that this will continue for some time. Since integrated STEM learning in German-speaking countries usually takes place in non-formal or informal settings via physical, face-to-face workshops, learning, and teaching STEM has been hit particularly hard.

The European project “MINT Learning Center (AB307)” aims to establish a network of STEM learning centres in Austria and Bavaria and thereby gives students the opportunity to discover and deepen STEM competencies in out-of-school settings with hands-on experiences using real-world problems. This project had to be fundamentally altered owing to COVID-19-related measures. Instead of offering face-to-face workshops to students in the STEM learning centres, our project had to migrate online. Despite this, we aspired to retain the same teaching and learning activities and processes in our online workshops. Organising these online activities also gave us opportunities for scientific research in which our goal was to identify potential success factors and stumbling blocks for conducting online STEM workshops in non-formal/informal learning settings. We also aimed to discover which elements of online learning or social distancing in STEM workshops should be continued in the post-COVID-19 era. In the process of achieving these research goals, our data collection and data analysis was guided by the following research questions:

- What are the potential success factors and stumbling blocks for conducting online STEM workshops in non-formal/informal learning settings from a teachers’ perspective?
- Which design elements of online STEM workshops in non-formal/informal learning settings that have proven successful during times of isolation or social distancing should also be used in post-pandemic times?

## Theoretical background

Integrated STEM learning practices offer a variety of opportunities and challenges for learners and teachers. However, this integration is often hindered by the rigidity of the educational system; by the unwillingness or fear of teachers to interconnect STEM subjects, or by the absence of STEM as a standalone subject (Portz 2015; Timms et al. 2018). Hsu and Fang (2019) state that in order to overcome challenges of learning and teaching STEM, pedagogies are needed for integrated STEM approaches and teachers equipped with STEM pedagogical content knowledge (PCK). PCK includes the pedagogical knowledge of teachers, their content knowledge, and how to use a combination of these for teaching and learning in school. What is unique about STEM PCK is that the teacher needs to be able to combine content knowledge from several subjects.

With the restrictions imposed by COVID-19, challenges for STEM teachers and STEM learning environments have increased even further and may be expected to apply especially to learning and doing STEM in non-formal/informal online or blended learning settings.

To better understand and classify teaching activities and the design of the STEM workshops in our research, we will now discuss learning in online learning environments (OLE) or blended/hybrid learning settings (BHLS), learning in non-formal or informal learning settings, and real-world or realistic problems in learning STEM.

### Learning in online or blended learning settings

As there are many descriptions and definitions of OLE and BHLE, we will first explain what we understand by OLE and BHLE. Following the systematic literature research by Singh and Thurman (2019), we interpret OLE as an environment in which technologies are used for synchronous and asynchronous learning and interaction at a physical distance. BHLE are environments in which the principles of OLE and brick-and-mortar learning environments are combined (Cahyono and Asikin 2019; Staker and Horn 2012). According to Tzu-Chi (2020), it is an advantage of technology-based learning environments like OLE or BHLE that spatial and temporal boundaries of learning environments are removed or softened.

Central to learning in OLE or BHLE are interactions between the participants themselves and between participants and the learning content, teachers and their competencies, and opportunities for personalising learning processes. According to Cummings et al. (2017) and Gillow-Wiles and Niess (2016), a lively interaction between the people involved and between the learners and the content is essential. Interactions of the participants should, on the one hand, increase the motivation and commitment of learners and, on the other hand, give teachers the possibility to flex their learning processes and to react to learners' requirements or problems. According to Zwart et al. (2017), an essential task of online teachers is to stimulate learners in such a way that they become cognitively active, discuss their ideas with each other, and thereby gain different perspectives on a problem. A major problem is that online teachers only know such learning environments and the resources and tools from a teacher's perspective (Gillow-Wiles and Niess 2016). This lack of learning experience concerning OLE or BHLE can result in content being too rigidly predetermined and thus not sufficiently responsive to learners’ needs (Tzu-Chi 2020). This rigidity of resources can lead to a loss of learners’ control which in turn may cause frustration or boredom for learners (Zwart et al. 2017). According to Adam et al. (2017), it is the control of one's own learning practices in OLE that could help learners to benefit. Another essential design pillar of OLE or BHLE is that the learning environment should be conveniently personalised (e.g. McGuire et al. 2017; Tzu-Chi 2020). Personalisation can include content in different formats, multiple uses of newly acquired knowledge or discussion platforms (Hsiao et al. 2019). Although learning in OLE or BHLE can also happen through interaction with the subject matter alone (e.g. watching learning videos or completing online quizzes alone), our project focused on participants’ interactions with the subject matter during interactions between the participants and the teacher. By aligning our project on learning through interaction between participants and the teacher, the teacher of the online STEM workshops should be able to respond to characteristics of participants, e.g. age, gender or expertise in the field, and related requirements, as well as to any content or technical problems participants may have. By responding to the requirements or problems of participants, a learning flow in online STEM workshops needed to be maintained.

### Learning in non-formal/informal learning

Learning in out-of-school contexts or in non-formal/informal learning environments presents abundant opportunities and challenges for both teachers and students. A typical feature of learning in such settings is that new knowledge and competencies are often developed hands-on (Mateos-Núñez et al. 2020) and then utilised (Radović and Passey 2016), resulting in concrete products (Tisza et al. 2019). These learning activities are also one of the central pillars of STEM workshops. STEM workshops with a focus on hands-on learning and developing concrete learning products should help prepare participants for lifelong learning, to use their competencies for solving everyday life or professional problems, and to increase learners' interest in STEM jobs (FitzSimons 2019; Navarro-Ibarra et al. 2017; Young et al. 2019).

Our STEM workshops were undertaken either at a specific location, or a location with special equipment (materials for the workshops) or location independant. Out-of-school learning activities at particular places or at places with special equipment are classified as part of non-formal learning according to the above experts. In informal learning, the place of learning is arbitrary. In terms of learning support, our STEM workshops display characteristics of non-formal as well as informal learning settings. It is typical for learning in a non-formal learning setting that support from a professional instructor is provided. In contrast, for learning in informal settings, support is usually provided by parents, friends, or peers. In terms of structure and organisation, workshops of our study could be described as mostly non-formal learning and characterised by a structured design (Radović and Passey 2016; Tisza et al. 2019). By conducting our non-formal or informal STEM workshops in the online space, specific challenges regarding the learning space and learning support arose. The particular location or equipment of a location was no longer laboratories and expensive machinery or toxic substances but, for example, participants' kitchens using baking powder or vinegar. Concerning the support of mainly younger participants by family members, in the online space teachers did not know whether students had home support or not. Conducting our workshops made it evident that in such settings, a high level of STEM PCK of the teachers was needed. This high level of teacher STEM PCK was needed, because there were many uncertainties or contingencies in non-formal or informal STEM workshops in the online space, to which the teacher has to react quickly and flexibly.

### Real-world or realistic problems in STEM learning

Integrating real-world problems into the learning of mathematics and the interdisciplinary application of mathematics—namely STEM—has positive effects on learners’ motivations, their understanding, and, above all, the comprehension of higher levels of the acquired content (Geiger et al. 2018; Ngiamsunthorn et al. 2016; Yang et al. 2017). Furthermore, according to Schukajlow et al. (2019), dealing with and solving real-world problems contributes to today's students being better equipped for their future lives. Ngiamsunthorn et al. (2016) add that using real-world problems in learning also implies that the context is multidisciplinary and that knowledge and competencies from different disciplines—in our case science, technology, engineering, and mathematics—are needed. Here, the aim is to relate students’ knowledge and competencies as a whole to real-world problems, thereby activating and making learners more active in learning (Afni 2020). These multiple benefits of using real problems have centuries-old traditions that are still relevant today (Larina 2016; Yilmaz and Ozyigit 2017). Despite these advantages and long traditions, school learning and teaching are still primarily oriented towards so-called real or real-world problems which are often only pseudo-realistic and have little in common with the lives of learners (Brown 2019; Smith and Morgan 2016; Yang et al. 2017). To take advantage of the benefits of using real problems and not to be limited to achieving curriculum goals or preparing for standardised testing, we have succumbed (exceeded?) replaced? school learning with our online workshops. To best integrate real-world problems into our online workshops, the focus was the use of real-world and tangible artefacts or the production of such artefacts. According to Bowen and Peterson (2019), Afni (2020) and Rach et al. (2018), including real-world problems in learning requires a comprehensive approach to learning, in which cross-curricular strategies and problem-solving skills are trained. Combining cross-curricular strategies and problem-solving skills is possible in our STEM workshops.

To combine the advantages of learning in online learning environments, non-formal or informal learning settings, and learning with and on real-world problems and to be able to investigate which design elements could support learning, we have developed, implemented, and scientifically investigated online STEM workshops in our study.

## STEM workshops

**Fig. 1:**
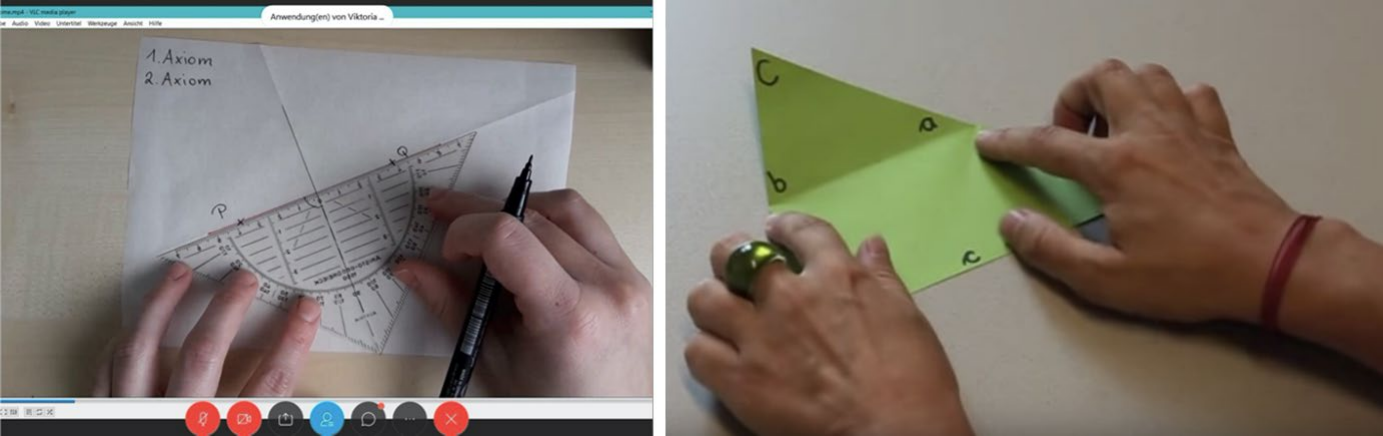
Photos from the Origami STEM workshop of our study. Links to the complete online STEM workshops: https://www.mintlabs.at/1-online-mintwoch/https://www.mintlabs.at/2-online-mintwoch/https://www.geogebra.org/m/dqsjvgbz

The STEM workshops were developed through and offered within the course of a cross-border project (MINT Learning Center, AB307) by four educational and research institutions from Austria and Germany. One of the objectives of the project was to develop extracurricular STEM learning opportunities for secondary school students (main target group lower secondary school, 10 to 14-year-old students). These extracurricular STEM learning opportunities were to be developed through cross-curricular and cross-institutional collaboration and offered to interested students through workshops. In developing STEM learning opportunities, a trans-disciplinary and cross-curricular approach was used. This approach was originally planned by developing STEM Learning Centres at the institutions of project partners. However, after starting the project in early 2020, the COVID-19 pandemic reached Central and Western Europe, making it obvious that the development of face-to-face STEM Learning Centres and conducting workshops there would not be feasible in the foreseeable future. For this reason, project partners decided to move our STEM workshops to the online space. Despite these changes and the relocation of the STEM workshops to online, a trans-disciplinary approach was continued. These workshops focused on real-world and tangible problems related to STEM. The topics and tasks of the online STEM workshops included building a solar oven, making cool-packs and moving a robot at a remote location (Fig. 1).

Between spring and autumn of 2020, STEM workshops were conducted both fully online and through technology extensions, while maintaining physical distance, in face-to-face form. Between 30 students per workshop and 170 students per workshop-day participated in these workshops which lasted from 30 to 45 min. The technology-enhanced, face-to-face workshops were offered in both guided and non-guided forms. The non-guided workshops took about 20 min but could be extended by participants as long as they wanted and were open to people of all ages. The guided workshops could be attended by 10 to 15 participants per workshop, lasted about 3 h and were open to students only.

## Methodological background

To identify potential success factors and stumbling blocks for online STEM workshops, and which design elements should be used in post-COVID-19 times, we conducted expert interviews with the teachers. The research data collected in our exploratory interview study was then evaluated, utilising principles of grounded theory approaches (GTA). As conducting online STEM workshops and related success factors have not yet been extensively researched, many experts (e.g. Charmaz 2006; Glaser and Strauss 1999) recommend using GTA to investigate the research topic.

### Grounded theory approaches

The professional network of teachers was at the centre of our research, for which many experts (e.g. Glaser and Strauss 1999; Mey and Mruck 2011) have used GTA. The professional network we investigated represents a small and limited section of reality, which has not yet been investigated in depth. For an investigation of such a small and limited selection of reality, using GTA was a suitable approach (Rosenkranz 2017; Strübing 2004). Specifically, we used the principles of a constructivist GTA (Charmaz 2006) in our research process. A constructivist GTA approach implies that the theories developed in the research process highly depend on researchers and their environments as well as on the teachers themselves. Since our study was intended to provide insights into a new area—online STEM workshops in non-formal settings which have not yet been studied in-depth—only a small part of the reality could be investigated. Due to this specific selection of the subject matter in our study, using constructivist GTA was deemed appropriate. Also due to the specific selection in our paper, it is the case that the insights gained by using GTA can only be utilised in the specific context of our study.

To achieve our research goal, data collection, data evaluation, and theory building were carried out in a cyclical process following GTA (Glaser and Strauss 1999; Kamin 2013; Mey and Mruck 2011). After each new interview, transcripts were coded and, using these new codes, existing theories were expanded. New theories then determined the focus of the subsequent data collection and data analysis. Following GTA, the dependence of subsequent data collection and data analysis on current theory corresponds to theoretical sampling (Glaser and Strauss 1999) and represents the guiding thread of our research steps. According to prominent representatives of the GTA (e.g. Charmaz 2006; Glaser and Strauss 1999), in the course of a GTA research process, everything can be data if it contributes to the achievement of the research goal. In addition to interview data, we have included data from participating and non-participating observations of the workshops and data on workshop materials in our theory development. We also reviewed and discussed the collected data and theories based on it with non-interviewed workshop teachers of our study, which should contribute to improving our theories.

### Data collection and participants

To achieve our research goal, we conducted data collection following the principles of GTA and qualitative interview studies (QIS). A total of eight interviews were conducted using online audio-visual tools. We decided to use online audio-visual tools to conduct our interviews as, on the one hand, to comply with the COVID-19 protection measures; on the other hand, to capture body language and emotion in addition to spoken words of participants. Our interviews were conducted over seven weeks and all interviews were recorded. During this seven-week period, we were able to integrate initial findings into our latter interview guides. The main topics of our interview guideline focused on (a) motivations and objectives of the online STEM workshops, (b) challenges and related strategies of conducting online STEM workshops, and (c) possibilities and opportunities of online STEM workshops. This timely integration of new findings into subsequent research activities was in line with using QIS and GTA and enhanced the quality of theories that emerged from our study.

The experts in our study were the teachers involved in our study who all had previous experience and expertise in planning and implementing STEM workshops. The task teachers was to plan, develop resources and then to conduct the workshops. The teachers were also asked to document any success factors or stumbling blocks. This would be used to plan and develop subsequent online or hybrid STEM workshops, as the European Union funded project is planned to run until summer 2022 and further workshops will be held. The teachers were aged in their mid-20s to early 60s. This range of teachers’ age enabled us to integrate expert perspectives of STEM workshop teachers into our theory development from the beginning, through the middle, to the end of their careers. This wide spread of age and experiences of participating teachers assisted us in facilitating and generalising our developed theories. The gender distribution of our teachers was mainly female: seven were women and one was a man, but this reflects the actual distribution of STEM teachers in Austria. The interviews lasted between 18 and 34 min—on average 26 min.

### Data analysis

To analyse the interview videos we used the principles of GTA and QIS. Following GTA, the techniques of open, axial, and selective coding, as well as theoretical sampling, were utilised (Charmaz 2006; Glaser and Strauss 1999). Generating initial codes, searching for themes, and reviewing themes following thematic analysis was in line with the QIS procedure (Braun and Clarke 2006; Ezzy 2002) and combined with the techniques of the GTA in our study.

In a first step, we watched the interview videos and tried to derive initial themes and patterns (Ritchie 2012) and later, interview videos were transcribed. Our initial video viewing formed the thread for open coding and generations of the initial codes. The aim of open coding and generating of initial codes was to break up the collected interview data into manageable analysis materials. These first codes had a low level of abstraction, and all codes were tagged. Below is a description and a prototypical extract from the transcripts (see Table 1).

**Table 1:** Prototypical take-out of an initial code from the code book

Keyword	Knowledge of the participants
Description	What knowledge do the participants bring with them/what do teachers take as basic knowledge
Interview-quote	Far more important than the age of the participants is their previous knowledge. I do not think that my 60-year-old mother knows more about physics than my 7-year-old son

In the next step, open codes were grouped according to similarities of definitions and text passages from the interviews. In this way, each coder was able to develop open codes with a higher level of abstraction. These more abstract codes then were compared between individual coders and the different codes were adjusted according to additional evaluations. In this way, the level of abstraction enabled additional intercoder reliability. This set of open codes was then used for axial coding (Table 2).

**Table 2:** Extract from the codes and categories at different levels of abstraction. Link to Code-Book: https://www.jku.at/fileadmin/gruppen/87/Sonstiges/CodeBook.pdf

Initial open codes	Codes with a higher level of abstraction	Core categories
Offers for pupils	Dissolving learning limits in terms of content	A new type of (social) interactions
Target group: old and young Audience is unknown	Preparation of interactivity and communication	Removing learning barriers and creating new types of learning places
Knowledge of the participants	Learning as a social act (at home and online)	Online socio-constructivist learning
Connecting/networking learners	Uncertainty in planning	
Groups of learners as a target group	Create products at home	Teachers' TPACK for STEM workshops
Dissolving thematic boundaries	Technologies lead to inclusion	

**Fig. 2:**
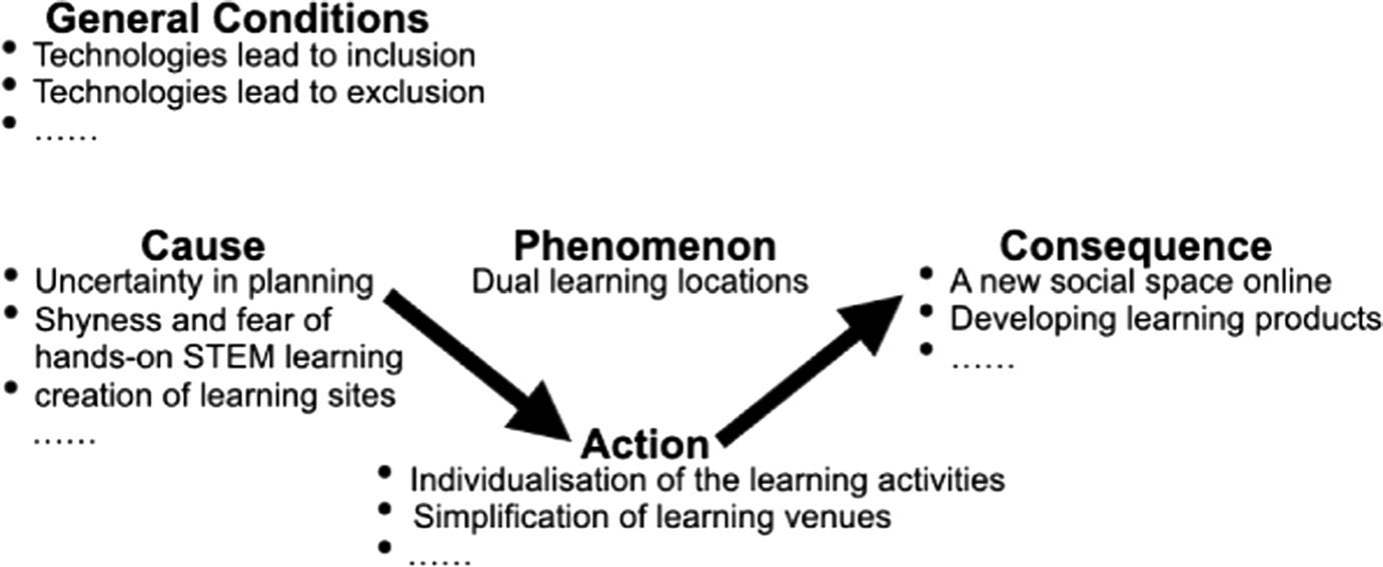
Axial coding

In the axial coding process, potentially central codes from open coding (phenomenon) were arranged to cause—action—consequence and general conditions (see Fig. 2). Code groups of cause—action—consequence when investigating the different phenomena were regrouped and tagged with new keywords. Our axial coding was aimed to create a synthesis of fragmented codes from open coding. These codes after axial coding were then used for selective coding.

Finally, selective coding aimed to identify connections and dependencies between the individual codes after axial coding and to find possible research gaps. Different coding principles were used after each interview and this cyclical process of data collection and data analysis led to hypothesis and question generation. Accordingly, interview guidelines and codebooks were continuously developed further during the project.

By using GTA and QIS, it became clear that for our workshop managers that the core categories were new types of (social) interactions, removing learning barriers, creating new types of learning places, online socio-constructivist learning, and teachers' TPACK for STEM workshops.

## Results

In the following, these core categories are outlined and explained with further details and quotes from interviews. The quotations from the interviews were translated from German into English by the research group and meta-information is provided alongside the quotations, accordingly our codes: M-male or F-female; based on teachers’ experiences, B—at the beginning of teaching career, M—in the middle of the career, and E—at the end of the career.

### New type of (social) interactions

Evaluating the interviews indicated that building our workshops on digital technologies resulted in experts considering technologies as door openers but also as gatekeepers. Technologies as door openers means that by holding workshops online, both in terms of geographical and social scope, and age focus, the workshops were made accessible to a broader audience than face-to-face workshops.

[F / M] *The online workshops allow us to reach more people. Anyone who wants to can take part, no matter where they live.*

For several experts in our study, addressing a broad audience was particularly important in STEM fields, as universities and other tertiary educational institutions have a strong interest in increasing the number of students studying STEM subjects.

[F / E] *It is always important for us to reach potential future students with the workshops and online it is much easier.*

On the one hand, increasing the potential audience was achieved by reaching students hundreds of kilometres away from the physical location of the workshop. On the other hand, many experts believed that such an online environment would reduce the scepticism or even fear of students and their parents from socially lower classes of universities in general and STEM studies in particular. Many experts believed that especially in the lower socio-economic classes of society, STEM is perceived as too difficult and therefore not considered to students’ further education and careers.

[F / B] *[…] you just have to click on a link. This makes it much easier for, say, students from simple backgrounds to participate … and then the parents can see that their children can do it, they can see that their children can be very talented in technology or science.*

Concerning teachers of our STEM workshops, the extended use of technologies in the workshops was seen as an opportunity. This opportunity was justified by the fact that one is not limited to experts from one's institution and that by holding our workshops online, teachers had more flexibility, which should lead to a larger number of experts willing to hold workshops.

[F / M] *Because the workshops could be held anywhere and at any time, it became much easier to encourage colleagues to offer their own workshops. This is usually a big problem.*

Technologies as a gatekeeper affected teachers and students of our online STEM workshops. In terms of learners, expert opinions indicated that potential bottlenecks of organising our workshops were the age of the learners and their socio-economic status. The age of learners, and especially the targeted use of learners' technologies associated with it by the experts, often led to problems and a loss of learners when expanding the online environment (e.g. opening a new website or installing additional online tools).

[F / E] *All participants had technical devices – otherwise, they could not have taken part. But especially with younger participants, it was the case that they could only look at the screen, if they had to do more, it felt like chaos.*

Following the statements of experts in our study, it was often the case that the students could not perform either these technical actions on their own or the students lacked the rights to do so. In terms of socio-economic status, workshop leaders of our study reported that it was often difficult to reach students from lower socio-economic backgrounds.

[F / B] *We actually took care that students do not have to install new software. But if students used certain tablets, they had to download an app and some younger participants had to find their parents to make this possible.*

This problem in reaching students from socio-economically lower classes could be explained by the fact that the technical framework conditions for students were not in place and/or the support of parents and the importance of education at home were not available. However, not only teacher-student interactions were changed by the online STEM workshops, but also learner interactions with STEM. Following feedback from our experts, face-to-face workshops at universities or research institutes also used high-end materials and machines. Students could also use these materials and machines on a temporary and voluntary basis in the face-to-face workshops. In addition, experts outlined that a small group of students became active in face-to-face workshops and wanted to interact with high-end materials and machines.

[F / M] *During the workshops in the laboratory, the students can take part in the experiment for a while, doing their own work. But often these particular machines, which are totally exciting for some, discourage others from taking part.*

By conducting our workshops online and thus within students' rooms and personal spaces, this high-end resource approach had to be abandoned. Instead of high-end resources and a small number of actively participating students, in an online space one had to use simple materials and all students were encouraged to participate to benefit from workshops.

[M / E] *If you didn't participate in the online experiment, well, you could have left it at that and just watch a video on the subject. So, the students had to participate in some way.*

This simplification was carried through the technological learning environments, materials, and also the content of the workshops. Simplifying learning leads to increasing hands-on learning of all students. In the opinion of our experts, this simplification also led to a more holistic teaching and learning process.

[F / E] *Due to the lack of laboratory materials, it was not possible to explore the topic as deeply as in face-to-face sessions, but one has to create tension in a different way—for example by demonstrating to students how things and processes of everyday life are scientifically connected.*

While face-to-face workshops often focused on a specific element or process or subject, the simplification of learning resources in our online workshop also led to investigating a phenomenon or process as a whole and therefore STEM became the centre of the learning process.

### Removing learning barriers and creating new types of learning places

The online STEM workshops took place in dual learning locations, meaning that places of teaching and places of learning could be geographically far apart from each other.

[F / B] *It was interesting to learn where the participants of the workshops came from. Some were even from another country.*

In addition to this geographical separation, it was also possible that teaching and learning locations were equipped with either the same resources or very different resources. Especially in the first phase of our online workshops, workshops were offered from teachers' kitchens or living rooms to students' kitchens or living rooms. Our experts believed that this equality between teaching location and learning location, and yet geographical separation of these two places, could represent a great learning potential of online STEM workshops.

[F / M] *That the students had the same materials to learn with as the teacher who led the workshop—this meant that students could do research just like real researchers, using simple materials.*

This framework led to teachers and students being able to perform the same learning or research activities. Thus, the learning of students corresponded to the actions of experts. This learning setting also meant that more students could be motivated to actively participate in learning processes in our STEM workshops.

[F / B] *Because the teachers of the workshop and the students of the workshop did exactly the same with the same materials, the enthusiasm of the students could be raised—at least with those who turned on the camera you could see that.*

A high number of active students was a significant advantage of online versus physical STEM workshops. Physical laboratory settings provide students with detailed insights into the daily work and research of scientists but often deter students from actually getting their hands on machines or materials.

[F / E] *So in the laboratory, only a few students really take part, now everyone had to actively participate because the oven did not build itself.*

In addition to teaching from teachers’ kitchen to the students' kitchen, holding online STEM workshops enabled teachers to offer them directly to students from laboratories. Although streaming out of laboratories had a positive effect on this form of workshop, nevertheless, the spirit of being in a laboratory was lost, which participants thought as special.

[F / E] *What you already lose in streaming is the spirit of a laboratory, actually being there and noticing that it also smells different there—that's what's missing in streaming.*

An advantage of streaming from laboratories was that workshops were available to any number of interested students, while face-to-face STEM workshops, like visiting a chemistry laboratory, are often reserved for only a few students. However, if workshops were streamed online from a laboratory, any number of students can participate.

[F / M] *Not so many students fit into or are allowed in the laboratory, sometimes not a whole class. This problem is solved by streaming.*

Another advantage of streaming from a laboratory was that the hazardousness of materials or experiments is no longer an obstacle.

[F / M] *For some, the chemistry laboratory is impressive, but for others it is frightening. If the students are already afraid of the laboratory and the materials, then they do not actively participate.*

Conducting our STEM workshops in an online space had increased the potential audience, as potential learning barriers from face-to-face workshops do not apply to online workshops. The two clearest barriers removed by on-line workshops were geographical distance and socio-economic background.

This also made it evident that learning can happen anywhere but can also be taught from anywhere. Depending on the topics of our STEM workshops or planned activities, online STEM workshops can be held from a high-quality laboratory to the teachers’ private kitchen.

### Online socio-constructivist learning

Although the majority of the STEM workshops in our study took place in an online environment and many students were alone at home in front of their digital devices, social-constructivist learning (i.e. developing knowledge and learning by social interaction) was a central element of the learning process.

[F / B] *Even though most of the workshops were held online, it soon became evident how important the social aspect is—especially when face-to-face events became possible again shortly before the summer, it became clear what had been missed in the past months.*

The social aspect of learning affected both teacher-students and student–student interactions. Concerning the joint learning processes of teachers and students, the feedback from our experts highlighted that social learning is essential for learning processes, but more difficult to initiate online than in face-to-face learning environments. To generate online social activities between teachers and students, it was important for our experts that teachers should initiate these learning activities.

[F / M] *In the online workshops, social learning or interaction does not just happen by itself as it can be in face-to-face workshops from time to time, the teacher has to do something to trigger it.*

Following the feedback of our experts, ways to trigger such online social actions between teachers and students could be to ask the learners questions directly or that learners present semi-finished or finished learning products including turning on their cameras. It was also important to our experts that in this context there were no universal processes for triggering teacher-students learning activities.

[F / E] *To trigger interactions with the learners, teachers need to prepare something. That they think about open questions or that there are online votes—whether this is successful depends on the group.*

This lack of standard procedures for initiating teacher-students cooperation would also apply to face-to-face learning settings. However, starting such cooperation in the online learning space would be much more difficult, because teachers do not know their students and/or have little or no information (age, level of knowledge, current participation in the STEM workshop, …) about students.

[F / B] *Since you usually do not know the group at all, neither before the workshop and you get to know them with difficulty during the workshop, many things become more difficult – especially everything that has to do with community.*

Although the majority of online STEM workshops were conducted without contact to other students at home, our experts indicated that social interactions among students were essential in online STEM workshops. Since, with few exceptions (e.g. breakout rooms in video conferencing), it was not possible to develop a learning environment that would allow students to work together in small groups on a project, other means of collaboration in online learning rooms had to be found.

[M / E] *What is always nice about face-to-face workshops is that students learn together, even if the students did not know each other before the workshop. This is almost impossible in the online space. We tried break-out rooms, but even that is certainly not ideal.*

Collaborative and social learning included students sharing finished or semi-finished learning products online. This online sharing of learning products could happen via closed learning environments of online STEM workshops or through social media channels. This online sharing of learning products aimed to inspire other students and to initiate possible new learning processes, to motivate other students, and also to create a sense of unity among our participating students.

[F / M] *To strengthen or develop a sense of community, we asked learners to share a photo of their slides and associated data through our social media platform. So that other people can think “Wow, this slide is fast” or “I know this slide, it is very close to my school.”*

A new form of social learning in online STEM workshops was cooperation and collaboration between parents and students or between family members in general. We were surprised and delighted that students from different age groups attended many of the online STEM workshops. According to our experts, this social form of learning was a novelty, which only became apparent when the STEM workshops were held online.

[F / M] *It was nice to see that at times a whole family joined in. From the child to the grandmother. And I think that the children and the grandma had the greatest joy.*

The fact that learning is an active and social process can also be clearly reflected in the feedback. However, this active and social process is probably more difficult to trigger in online spaces than it is in face-to-face settings. Teachers could have a key role to getting such activities and social action into online learning of STEM workshops.

### Teachers’ TPACK (technological, pedagogical content knowledge) for STEM workshops

Essential design elements of online STEM workshops were dissolving learning boundaries, activating students or triggering socio-constructivist learning activities in online spaces. To develop and use these design elements for online learning spaces, feedback from the experts in our study indicated that teachers of online STEM workshops need to have in-depth technological, pedagogical content knowledge (TPACK).

[F / M] *Even more important than usual is that the teachers and the knowledge and also the enthusiasm of the teachers are extremely important for the workshops.*

A central element that required the expertise of teachers of our online STEM workshops was the uncertainty regarding the number of participants of the individual workshops. Since no registration was required to participate in the online STEM workshops, teachers did not have any information in advance about the number of participants in the individual workshops, the age of the participants, and the professional and technological background of the participants.

[F / M] *We have chosen not to require registration in order to avoid creating yet another hurdle for participants. However, this meant that we did not know whether or how many learners would come to the workshop, nor what knowledge the participants had or what interests they had.*

This uncertainty regarding the participants meant that teachers were required to be highly spontaneous and have expertise in the areas of knowledge, use of technologies, and implementation of teaching–learning approaches. The need for spontaneity and high expertise of the workshop teachers was reinforced by the fact that the group of students in the respective online STEM workshops did not remain constant.

[F / B] *You have to be able to be spontaneous in workshops. In the online workshops, it happened that people suddenly left the room or that new people were in the room again—this increased the need for spontaneity.*

Non-constant groups of students in the online STEM workshops implies that during the workshops, students have also left the workshop, and new students have also joined during the workshop. This change of participants could also change the needs and requirements of the students or the dynamics of the student group. In order to maintain a learning flow despite these changes, the teacher needed to have in-depth expertise in the areas of content, technologies and online teaching and learning approaches to be able to react spontaneously.

[F / B] *If the group changes too much during the workshop, things or explanations that worked well before suddenly stop working and you have to react quickly, or you will lose your participants.*

Another major challenge for teachers of online STEM workshops was to trigger interaction and social learning in the workshops. On the one hand, triggering of interactions and social learning in the online STEM workshops had to be planned, as interaction and social learning do not develop by themselves.

[F / M] *You need different strategies and means to involve the participants in the workshops. We wrote questions in the chat or asked open questions to the participants during the workshops. With others we have seen that you can also use surveys or multiple-choice questions during the workshop, we will try that next time.*

On the other hand, the uncertainty of our participants required different triggers to be used for different groups of students. These different triggers had to be planned and developed in advance and then used in a targeted manner in this situation. Both planning and implementing, as well as the situation-specific selection of the conditional triggers, required a profound knowledge of the teachers of the online STEM workshops.

[F / M] *What helps in triggering learning or interaction in online workshops is that you have experience of what works in face-to-face workshops. You cannot take these strategies on one-to-one, but you can adapt it to the specific situation.*

The provision of opportunities for individualisation of the online STEM workshops, both during and especially after workshops, required a high level of expertise from our teachers. Most teachers wanted to provide further learning opportunities for their students. As students were able to use these learning opportunities at any time and as the previous knowledge and age of the students were unknown, these additional learning opportunities had to be created with ample pedagogical and technological sophistication.

[F / M] *We also wanted the learners to be able to deal with the content on their own. But to do this we needed a platform that would allow us to link the material of the different subjects. I did not find such a platform—I had to create a website myself—but that is only possible because I can create websites “by chance”.*

Technological, pedagogical, and content competencies or TPACK, and combinations of these competencies, could be even more important for teachers of online STEM workshops than for other teachers. This can be explained, by the fact that STEM consists of four subject areas and their combination. This requires rich content knowledge. In non-formal/informal online workshops there is usually no prior information about the group of learners and the groups of learners are mostly heterogeneous. To deal with this uncertainty high pedagogical competencies are needed.

In the course of our study, it was shown that technologies can act as both a door opener and a gatekeeper. Depending on how and which technologies and other resources are used per target group of online STEM workshops, this can lead to an increase or decrease of the group of potential participants and possibilities for activity of the participants. Using well-selected technologies and other resources could also lead to temporarily abstract concepts of STEM being easier or better connected to the real environments of the students. This easier or better connection of STEM concepts and students' real environments could also lead to more authentic learning. Authentic learning, even if it takes place mainly via digital communication technologies and spatially separated students and teachers, is according to the results of our study to a high degree a social act. In order to make learning with digital communication technologies and spatially separated students and teachers a social act, it is important that there is a synergy of technologies and pedagogies. For these to be combined fruitfully, good and well-trained teachers with extensive TPAC knowledge are needed.

## Discussion

In exploring the conditions for success in conducting online STEM workshops in non-formal/informal learning settings, we discovered that certain design elements of STEM workshops should also be used in post-COVID-19 times. These design elements include offering new types of (social) interactions, removing learning barriers, creating new types of learning places, online socio-constructivist learning, as well as teachers' technological, pedagogical, and content competencies.

For the experts of our study as well as in other recent studies (Tzu-Chi 2020), it was shown that the removal of spatial and temporal boundaries is a significant advantage of OLE or BHLS. In addition, our study also indicated that OLE or BHLS, as used in our online STEM workshops, have the potential to unify learning spaces so that students could benefit from and use the equipment of physical classrooms or laboratories at home. We employed such equipment in our online STEM workshops by allowing students to control the movements of mini-robots in a remote laboratory from their computers at home.

Also creating learning products when the place of learning and the place of teaching or the laboratory were spatially separated was an essential part of our online setting. Hence, our study indicates that creating learning products—an essential aspect of active or hands-on learning of STEM, which according to Mateos-Núñez et al. (2020) and Tisza et al. (2019) is also crucial in face-to-face settings—can also be done in online environments.

In our study, 3D printing was used to produce concrete learning products and thereby combine physically separated learning and teaching location. The objects were designed in students' living rooms and realised or brought to reality in remote teachers’ laboratories.

According to Cummings et al. (2017) and Gillow-Wiles and Niess (2016), one of the essential elements of learning in OLE or BHLS is that there is a lively interaction when learning. This interaction concerns both the interaction of all participants and the interaction of learners with the fields of knowledge to be learned and researched. In our study, we confirm this importance of interaction in online environments. Furthermore, our study indicated that triggering and creating interactions could be particularly challenging because our participants were unknown to each other. On the one hand, the learners in a digital environment do not need to know each other per se. On the other hand, teachers do not need to know who their audience is and therefore, which means could trigger interactions that might be promising Meaning?

As already mentioned in the theoretical framework, feedback from the experts of our study also showed that a dichotomy between learning in non-formal and informal learning settings (Radović and Passey 2016; Tisza et al. 2019) is only partially applicable to the online STEM workshops of our study. Although a variety of elements of non-formal learning settings, such as a professional teacher or specific materials for learning (e.g. baking powder or aluminium foil) were also relevant in our study, some elements of typical informal learning settings were found as well. Typical elements of informal learning in our study are that support from parents or other family members was essential especially in the post-phase of the online STEM workshops the students could choose further learning paths and activities on their own. According to the findings of our study, it might therefore be useful to investigate whether the division between non-formal and informal learning settings can also be maintained in online learning settings.

According to Zwart et al. (2017), a joint discussion of ideas and solution strategies, and thus interactions of learners is an essential pillar of OLE or BHLS. This joint discussion of ideas and solution strategies can be characterised as cooperative learning. In our study, we found that online STEM workshops could take different approaches to learner interactions, especially in non-formal or informal settings. According to Zwart et al. (2017), it is cooperative learning which should activate students and trigger learning. However, feedback from our experts indicates that in online STEM workshops, in non-formal or informal settings, it could be competitive learning that activates and motivates the students. This competitive learning was realised in the online STEM workshops of our study by asking workshop participants to upload performances (e.g. photos of slides and associated data) or to upload photos of finished learning products in the social media channels of workshop providers and to generate Likes.

A key factor in getting students into online STEM workshops to learn actively and interact with others or the subject to be learned could be the teachers of the workshop, according to the results of our study. A problem of teachers in OLE or BHLE, according to Gillow-Wiles and Niess (2016), is that they know OLE or BHLE only from the teachers’ perspectives. This lack of experience from students’ perspectives could lead to teachers having problems to put themselves in students' shoes, which was also reflected in the feedback from the experts in our study. STEM, or more precisely STEM teachers, could be another challenge or problem. Hsu and Fang (2019) emphasise the importance of specific STEM PCK. However, results of our study show that it is not just a combination of STEM and PCK, but rather a combination of STEM and TPACK (Mishra and Koehler 2006) that teachers of STEM workshops need. If STEM workshops are offered in an online setting, the T of TPACK for teaching STEM should be emphasised.

## Conclusions

A new type of (social) interactions, removing learning barriers and creating new types of learning places, online socio-constructivist learning, and teachers' TPACK for STEM workshops were the core categories for success in conducting and designing online STEM workshops in non-formal learning settings. These core categories could also contribute to improving the quality of STEM workshops in post-COVID-19 times.

A central aspect of our study, which may be equally important in the future, is that less or simpler materials adds more benefits for students and make them available in students’ homes. If these materials are not yet available, materials should be easy to obtain, inexpensive, and students should be familiar with the resources. Integrating such simple materials into future STEM workshops leads to additional motivation for students. Can reduce students’ shyness in online environments and increase the number of students actively participating in STEM workshops.

The use of a variety of appropriate technologies in STEM workshops also contributes to bringing together geographically separated spaces of teaching and learning. By bringing together teaching and learning spaces, the audience of STEM workshops is enlarged, and more inclusion achieved. This inclusion extends to students from rural areas or regions far away from available STEM workshops. Inclusion also supports students from lower socio-economic backgrounds, as the barrier to participating in online STEM workshops is lowered by combining teaching and learning spaces.

Active learners and a larger and more mixed audience means that a high level of expertise is required from teachers of STEM workshops. It might be helpful if this high level of expertise is provided not only by one teacher but by a pair or a team of teachers. In STEM, especially in online STEM workshops, teachers' expertise in TPACK is likely to be particularly important. The high importance and the requirement for C (content) in STEM workshops is evident, as expertise from up to four scientific fields should be combined into one workshop. However, by conducting STEM workshops in OLE or BHLE, T (technological) and TP (technological and pedagogical) also have an exceptionally high significance. On the one hand, teachers need expertise in T to develop OLE or BHLE. On the other hand, a high level of TP is required by teachers to trigger interactions in STEM workshops in digital environments. This need for high expertise in TP could be increased if STEM workshops are part of non-formal or informal learning.

## Limitations

Although our study was conducted for several months, and we tried to include a wide range of experts in our study, some elements limit our results and implications of our findings. The first limitation concerns framework of our study—lockdowns, social distancing, and insecurity in broad sections of society due to the COVID-19 pandemic. A further limitation concerns the basic technological equipment in the region of our study. Although experts of our study reported clear inequalities in the availability of technological resources, our study took place in an area of high socio-economic standard and the minimum level of technological equipment in this region exceeds the average technological equipment of many other regions. A further limitation results from the administrative and educational framework of our study. The four organisations involved in the development of the STEM workshops have a high level of pedagogical and technological expertise and years of experience in the development of STEM workshops. By providing additional funding through the European Interreg project “MINT Learning Centern—AB307”, this experience and expertise of the four organisations could be put to best use.

## Data Availability

The datasets used and analysed during the current study are available from the corresponding author on reasonable request. Please contact the author for data requests. The Code Book can be found online: https://www.jku.at/fileadmin/gruppen/87/Sonstiges/CodeBook.pdf.
